# Private Correspondence

**Published:** 1868-10

**Authors:** 


### PRIVATE CORRESPONDENCE.

What a pile of unanswered letters! How unfriendly it was not to answer them! "But if a man couldn't how could he?" No doubt that very many of our professional brethren feel slighted because we failed to answer their many inquiries. We simply could not *spare* the nerve force to do so. We have not neglected their wishes more than we have our own business. We hope this explanation will relieve any feeling of suspense pertaining to the matter. Not many will believe their letters have been neglected for want of friendship or lack of interest. Though not expecting to abound in private correspondence, we hope to contribute more to the pages of the REGISTER than for a few months back; but even of this it is not best to be too confident. W.

